# Functional assessment of coffee cherry-derived non-conventional yeasts identifies promising candidates for probiotic and postbiotic applications

**DOI:** 10.3389/fmicb.2026.1920410

**Published:** 2026-08-12

**Authors:** Victor Cifuentes, Gratiela Gradisteanu Pircalabioru, Gabriela N. Tenea

**Affiliations:** 1 Biofood and Nutraceutics Research and Development Group, Faculty of Engineering in Agricultural and Environmental Sciences, Universidad Técnica del Norte, Ibarra, Ecuador; 2 Department of Botany and Microbiology, Faculty of Biology, University of Bucharest, Bucharest, Romania

**Keywords:** antimicrobial activity, biosafety, coffee fermentation, functional foods, *Kurtzmaniella quercitrusa*, *Meyerozyma caribbica*, non-conventional yeasts, postbiotics

## Abstract

**Introduction:**

Non-conventional yeasts (NCYs) associated with spontaneous food fermentations represent an underexplored source of microorganisms with potential probiotic and biotechnological applications. Coffee cherry fermentations harbor diverse yeast communities; however, the functional properties of indigenous NCYs remain poorly characterized. This study identified NCYs isolated from coffee cherry fermentations and evaluated their probiotic-associated functional traits, including stress tolerance, biosafety, antimicrobial activity, cytocompatibility, and effects on epithelial cell migration.

**Methods:**

Five NCY isolates were identified by ITS rDNA sequencing and characterized using *in vitro* assays evaluating tolerance to simulated gastrointestinal conditions, osmotic and thermal stress, cell surface hydrophobicity (CSH), autoaggregation, extracellular enzyme production, hemolytic activity, susceptibility to antifungal agents, and antimicrobial activity of neutralized cell-free supernatants (CFS). Cytocompatibility of CFS was assessed in Caco-2 cells using LDH, MTT, and Live/Dead assays, whereas epithelial cell migration was evaluated using a scratch assay.

**Results:**

Four isolates were identified as *Kurtzmaniella quercitrusa*, whereas one isolate was identified as *Meyerozyma caribbica*. All isolates exhibited strain-dependent tolerance to simulated gastrointestinal and environmental stresses. Lev3 showed the highest tolerance to gastrointestinal conditions, with survival comparable to the reference probiotic *Saccharomyces boulardii* CNCM I-745 (SSb), whereas Lev10 exhibited superior tolerance to osmotic and thermal stress. CSH and autoaggregation were also strain dependent, with Lev10 and Lev5 exhibiting the most favorable adhesion-related profiles among the coffee-derived isolates. All isolates were catalase-positive and exhibited variable proteolytic activity. None showed hemolytic activity, and most were susceptible to fluconazole and nystatin. Neutralized CFS exhibited inhibitory activity against both Gram-positive and Gram-negative bacteria while displaying low cytotoxicity toward Caco-2 cells. Although CFS did not significantly enhance scratch closure relative to the untreated control, Lev11 and Lev13 maintained epithelial wound closure comparable to the control.

**Conclusion:**

Coffee cherry-derived NCYs exhibited strain-dependent probiotic-associated traits, favorable preliminary biosafety profiles, antimicrobial activity, and high cytocompatibility, supporting their potential for fermentation biotechnology and the development of next-generation probiotic or postbiotic products. Further genomic, metabolomic, and *in vivo* studies are required to validate their efficacy, safety, and mechanisms of action.

## 1 Introduction

Yeasts are important contributors to food and beverage fermentations, with *Saccharomyces cerevisiae* var. *boulardii* representing the best-characterized probiotic yeast (Pereira et al., 2025). Unlike bacterial probiotics, yeasts exhibit intrinsic resistance to antibacterial agents, enabling their concurrent use during antibiotic therapy (Hatoum et al., 2012). In addition to their tolerance to gastrointestinal conditions and interaction with host immune responses, many yeasts produce extracellular antimicrobial compounds that influence microbial ecology and contributed to food preservation. In addition, yeasts produce antimicrobial peptides and related compounds that influence microbial dynamics and contribute to biocontrol. For instance, *S. cerevisiae* CCMI 885 secretes low-molecular-weight peptides active against spoilage and pathogenic yeasts, including *Hanseniaspora, Torulaspora, Kluyveromyces, Lachancea*, and *Brettanomyces* spp. (Albergaria and Arneborg, 2016; Muccilli and Restuccia, 2015). Similarly, a ~ 5 kDa peptide from *Candida intermedia* inhibits *Brettanomyces bruxellensis* (Al-Sahlany et al., 2020), while killer toxins produced by *S. cerevisiae* display antimicrobial activity against *Escherichia coli, Salmonella* spp.*, Staphylococcus aureus, Pseudomonas aeruginosa*, and *Candida albicans* (Kumura et al., 2004; Hatoum et al., 2012). Together, these findings highlight yeasts as promising sources of antimicrobial compounds for microbial control and food preservation.

Recent advances in microbial biotechnology have intensified interest in non-conventional yeasts (NCYs), which are frequently isolated fruits and fermented foods and beverages (Tamang et al., 2016; Ye et al., 2019; Melini et al., 2021; Martins et al., 2025). Genera such as *Meyerozyma, Pichia*, and *Torulaspora* include species with diverse functional properties, including antimicrobial and antioxidant activities, gastrointestinal stress tolerance, and modulation of gut microbial communities (Arévalo-Villena et al., 2020). Among these, *Meyerozyma caribbica* and *Kurtzmaniella quercitrusa*, formerly classified within the polyphyletic genus *Candida,* are commonly associated with fruit fermentations and have attracted growing interest (Piskur and Langkjaer, 2004; El-Adawy et al., 2020). *M. caribbica* exhibits several probiotic-associated traits, including tolerance to acidic and bile conditions, auto-aggregation, adhesion to epithelial cells, antimicrobial activity, and antioxidant capacity (Amorim et al., 2018; Padilha et al., 2018; de Moraes et al., 2021; Simões et al., 2021). In contrast, *K. quercitrusa* remains largely unexplored, despite evidence of metabolic adaptability across diverse environments.

Beyond the effects of viable cells, NCYs may produce extracellular metabolites with biological activities that could contribute to host- or process-related benefits and therefore warrant investigation as potential postbiotic preparations. Yeast-derived compounds, including phenolic molecules, glutathione, vitamins, organic acids, and antioxidant enzymes such as superoxide dismutase and catalase, contribute to cellular protection against oxidative stress, a process associated with chronic disease development (Zago et al., 2022; Reddy, 2023). These functional properties are highly strain dependent and reflect differences in metabolic output, antimicrobial activity, and antioxidant potential. Consistent with this variability, microbial consortia isolated from fermented *Coffea arabica* cherries exhibited substantial heterogeneity in metabolite production and bioactivity among strains, including selected NCYs with promising functional characteristics (Cifuentes et al., 2025).

Increasing evidence indicates that yeast-derived extracellular metabolites may exert biological activities extending beyond antimicrobial effects, including modulation of epithelial integrity, oxidative stress, and tissue repair (Tadioto et al., 2023). Several NCYs have been investigated as microbial platforms for producing bioactive molecules with potential applications in biotechnology and regenerative medicine. For example, *Yarrowia lipolytica* synthesizes lipids, organic acids, and enzymes that have been explored for tissue repair and wound healing applications (Fickers et al., 2020; Lee et al., 2025). These findings support evaluating the biological activity of yeast-derived cell-free supernatants in epithelial models.

Despite increasing interest in NCYs from fermented foods (Staniszewski and Kordowska-Wiater, 2021; Yan et al., 2021), limited information is available regarding the functional characteristics and bioactive extracellular metabolites of yeasts isolated from fermented *C. arabica* cherries. Accordingly, the present study evaluated the probiotic-associated traits and biological activities of five NCYs isolated from three fermented *C. arabica* cherry varieties from Intag, Ecuador. The study included assessment of tolerance to simulated gastrointestinal conditions, osmotic and thermal stress, cell surface properties (hydrophobicity and autoaggregation), biosafety-related characteristics, and antimicrobial activity of extracellular metabolites present in yeast-derived cell-free supernatants (CFS). Their biological activity was further assessed through cytotoxicity and scratch assays using Caco-2 cells to evaluate epithelial cell compatibility and restitution. Together, these analyses aimed to identify strain-dependent functional traits and extracellular bioactive products with potential applications in food biotechnology and the development of next-generation probiotic candidates and postbiotic preparations.

## 2 Materials and methods

### 2.1 Isolation and selection of yeasts

Yeast isolates were obtained from spontaneous fermentations of coffee cherries conducted for 6 days at room temperature. Cherries were collected at two ripening stages (immature green and mature red/ yellow) from three *Coffea arabica* varieties: Typica (TYP), Yellow Caturra (CATY), and Red Caturra (CATR). The physicochemical characteristics and yeast populations of the fermented cherries are presented in Supplementary Table S1. Serial dilutions of the fermented cherries were plated on yeast extract peptone dextrose (YPD) agar, and colonies exhibiting distinct colony characteristics (e.g., size, shape, and color) were purified and examined by light microscopy to confirm yeast morphology. The purified isolates were subsequently screened for fermentative capacity (highest CO₂ production) in YPD broth. When multiple colonies with similar morphological characteristics were recovered from the same sample, only one representative isolate was retained to avoid duplicate characterization. Consequently, one representative isolate was selected from each variety-ripening stage. No yeast colonies were isolated from fermented immature green cherries of Red Caturra, consistent with the absence of detectable viable yeasts in this sample (Supplementary Table S1). The five selected isolates were designated “Lev” (from the Spanish word *levadura*, meaning yeast): TYPVLev13 (Lev13), isolated from fermented immature green Typica cherries; TYPRLev10 (Lev10), from mature red Typica cherries; CRYVLev5 (Lev5) and CRYALev3 (Lev3), from fermented immature, green and mature yellow, Yellow Caturra cherries, respectively; and CATRLev11 (Lev11), from fermented mature red Red Caturra cherries. Cultures were preserved in YPD broth supplemented with 20% (w/v) glycerol at −80 °C and reactivated in fresh YPD medium prior to further analyses.

### 2.2 Identification of selected isolates

Genomic DNA extraction, polymerase chain reaction (PCR) amplification, and sequencing were performed by Macrogen Inc. (Seoul, Korea) following their standard protocols. Molecular identification was performed by PCR amplification of the internal transcribed spacer (ITS) region, encompassing the 5.8S rRNA gene. Amplification was carried out using the universal primers ITS4 (5′-TCCTCCGCTTATTGATATGC-3′) and ITS5 (5′-GGAAGTAAAAGTCGTAACAAGG-3′). Sequencing reactions were conducted using the PRISM BigDye™ Terminator v3.1 Cycle Sequencing Kit (Applied Biosystems). The extension products were combined with Hi-Di™ formamide (Applied Biosystems, Foster City, CA, United States), denatured at 95 °C for 5 min, rapidly cooled on ice for 5 min, and analyzed on an ABI Prism 3730XL DNA Analyzer (Applied Biosystems). Putative species-level identification and phylogenetic placement were achieved by comparing the obtained sequences against the National Center for Biotechnology Information (NCBI) database using the nucleotide Basic Local Alignment Search Tool (BLASTn).

### 2.3 Phylogenetic analysis

The ITS-rRNA gene sequences obtained from the yeast isolates were compared with reference sequences retrieved from GenBank to determine their taxonomic affiliation. Reference sequences corresponding to *K. quercitrusa* (PP589185), *Candida quercitrusa* (KF747756), and *M. caribbica* CBS 9966 were included to support species-level identification. The ITS sequence of *Hyphopichia* sp. (GenBank accession no. ON508865) and *Pichia caribbica* (GenBank accession no. FN428887) were included as an outgroup to root the phylogenetic tree. Sequences were edited and assembled using MEGA v.11 (PSU, United States). Multiple sequence alignment was performed using the MUSCLE algorithm with default parameters. Phylogenetic relationships were inferred using the Neighbor-Joining (NJ) method based on the Kimura two-parameter (K2P) nucleotide substitution model. Branch support was assessed by bootstrap analysis with 1,000 replicates, and bootstrap values greater than 50% are shown at the corresponding nodes. Branch lengths represent the estimated number of nucleotide substitutions per site.

### 2.4 Probiotic-associated traits evaluation

Three reference yeast strains were included in the probiotic-associated traits assays: the commercial probiotic strain *S. boulardii* CNCM I-745 (SSb), and two laboratory strains *Saccharomyces cerevisiae* Lev30 (Lev30), and *Candida intermedia* Lev9 (Lev9). The selection of reference strains depended on the experimental endpoint evaluated and is described in the respective assay.

#### 2.4.1 Tolerance to modified simulated gastric juice and bile salts

Tolerance to modified simulated gastric conditions and bile salts was assessed as previously described by Tenea et al. (2022). Yeast cultures were centrifuged at 5,000 × g for 5 min at 4 °C, washed twice with sterile Ringer’s solution (pH 7.2), and resuspended in simulated gastric juice adjusted to pH 2.5 and pH 3.0. Suspensions were incubated at 37 °C for 4 h, and viability was determined hourly by plating on YPD agar. The simulated gastric juice contained glucose (3.5 g/L), NaCl (2.05 g/L), KH₂PO₄ (0.60 g/L), CaCl₂ (0.11 g/L), KCl (0.37 g/L), pepsin (3 g/L), and lysozyme (0.1 g/L), according to Corcoran et al. (2005). For bile tolerance, overnight cultures (1 × 10^8^ CFU/mL) were incubated in YPD broth supplemented with 0.3 and 1% (w/v) oxgall bile salts at 37 °C for 4 h. Cell survival was calculated as follows: survival (%) = (CFU final / CFU initial) × 100. The probiotic reference strain SSb was included for comparison. All assays were performed using independent biological triplicates.

#### 2.4.2 Tolerance to sodium chloride and temperature

Sodium chloride tolerance was evaluated by culturing yeast strains in YPD broth containing 1 and 4% (w/v) NaCl. Cultures were incubated at 37 °C for 24 h, and growth was evaluated by viable plate counts on YPD agar. Temperature tolerance was assessed at 4, 37, and 45 °C under identical conditions. Survival percentages were calculated as described in Section 2.4.1. Unmodified YPD broth served as the control. The probiotic reference strain SSb was included for comparison. All assays were performed using independent biological triplicates.

#### 2.4.3 Cell surface hydrophobicity (CSH) and autoaggregation

CSH was determined according to the microbial adhesion to hydrocarbons method described by Shazad et al. (2025). Yeast suspensions (1 × 10^8^ CFU/mL) were prepared in phosphate-buffered saline (PBS; pH 7.2) following centrifugation at 5,000 × g for 5 min at 4 °C. The initial absorbance (A₀) was measured at 600 nm. Cell suspensions were mixed with 1 mL of hexane, ethyl acetate, or chloroform and vortexed for 1 min. After phase separation (10, 30, 60, and 180 min), the absorbance of the aqueous phase (*A*₁) was measured. Hydrophobicity was calculated as follows: Hydrophobicity (%) = [1-(A₁/*A*₀)] × 100. Autoaggregation ability was evaluated using a modified method described by Shazad et al. (2025). Yeast cells (1 × 10^8^ CFU/mL) were washed, resuspended in PBS (pH 7.2), vortexed for 10 s, and the initial absorbance (*A*₀) was measured at 600 nm. Samples were incubated at 37 °C and absorbance values were recorded at 2, 4, 6, 8, and 24 h. Autoaggregation was calculated as follows: Autoaggregation (%) = [1 − (A_t_/A_0_)] × 100 (Shazad et al., 2025). The results were compared with the reference strains SSb, Lev30, and Lev9. Mean values of hydrophobicity and autoaggregation were subjected to hierarchical cluster analysis using Euclidean distance as the similarity metric and complete linkage as the clustering algorithm (Borcard et al., 2018). The resulting dendrograms and heatmaps were used as exploratory tools to visualize similarities in adhesion-related phenotypes among strains rather than for hypothesis testing or inferential statistical analysis.

#### 2.4.4 Qualitative protease and catalase activity

Extracellular protease production was assessed following the method described by Hanane et al. (2022). Yeast isolates were inoculated onto YPD agar supplemented with 10% skim milk powder and incubated at 30 °C for 7 days. Proteolytic activity was indicated by the formation of a clear halo surrounding colonies due to casein hydrolysis. Catalase activity was determined by adding 3% (v/v) hydrogen peroxide directly onto 48 h-old yeast cultures (Hanane et al., 2022). Immediate bubble formation was considered indicative of catalase activity. Results were compared with SSb, Lev30, and Lev9.

### 2.5 Biosafety evaluation

#### 2.5.1 Hemolytic activity

Hemolytic activity was evaluated using Columbia agar supplemented with 5% sheep blood according to Yasmin et al. (2020). Plates were incubated at 37 °C for 48 h, and hemolytic patterns were classified as *α*-hemolysis (greenish halo), β-hemolysis (clear halo), or *γ*-hemolysis (absence of halo). Strains exhibiting γ-hemolysis were considered non-hemolytic. Results were compared with SSb, Lev30, and Lev9.

#### 2.5.2 Susceptibility to antifungal agents

Susceptibility to antifungal agents was evaluated using an agar well diffusion assay following the CLSI M44 disk diffusion guideline (Clinical and Laboratory Standards Institute [CLSI], 2018). Overnight yeast cultures were spread uniformly onto YPD agar plates. Wells were aseptically prepared using a sterile cork borer and filled with antifungal solutions containing amphotericin B (32 μg/mL), voriconazole (2 μg/mL), fluconazole (64 μg/mL), or nystatin (1.25 μg/mL) (Merck, United States). Plates were incubated at 37 °C for 48 h, after which inhibition zones were measured in millimeters using an automatic plate scanner (Scan500, Interscience, France). Larger inhibition zones were interpreted as indicative of greater antifungal susceptibility under the tested conditions. All assays were performed using independent biological triplicates.

### 2.6 Cell-free supernatant (CFS) characterization

#### 2.6.1 CFS extraction

An overnight yeast culture was inoculated into 100 mL of YPD broth supplemented with 10% sucrose and incubated at 30 °C for 48 h with agitation at 200 rpm. The medium was supplemented with sucrose because elevated sugar concentrations have been reported to enhance the production of extracellular bioactive metabolites by several yeast species (Tenea et al., 2023), thereby increasing the recovery of compounds for CFS characterization. Cultures were centrifuged at 13,000 × g for 20 min at 4 °C, and the resulting supernatants were sequentially filtered through 0.45 and 0.22 μm syringe filters (Chemlab Group, Washington, DC, United States). Filtered CFS samples were lyophilized and stored until analysis.

#### 2.6.2 Antimicrobial activity toward bacterial pathogens

Antimicrobial activity was evaluated using the indicator bacterial strains: *Escherichia coli* ATCC 25922, *E. coli* L1PEag1, *Staphylococcus aureus* ATCC43300, *S. aureus* ATCC1026, *Salmonella enterica* ATCC51741, *S. enterica* ATCC35640, *Shigella sonnei* ATCC25931, *Enterobacter* sp. dFMcEag13, *Kluyvera intermedia* dfMPEag15, *Ewingella* sp. UMEag21, *Pantoea* sp. UMEag23, *Serratia liquefaciens* P4StpC1, *Listeria monocytogenes* ATCC18115, *S. epidermidis* L4MStp5, *S. xylosus* FFCShyA4. Bacterial suspensions (1 × 10^7^ CFU/mL) were mixed with 0.75% nutrient soft agar and overlaid onto nutrient agar plates. Prior to testing, yeast-derived CFS were adjusted to pH 7.0 using sterile NaOH to eliminate the inhibitory effect associated with organic acids and low pH. Wells (6.5 mm diameter) were aseptically prepared in the agar and filled with 100 μL of neutralized CFS. Plates were incubated at 37 °C for 24–48 h, after which inhibition zones were measured using an automatic plate scanner (Scan500, Interscience, France). All assays were performed in triplicate (independent biological replicates). YPD broth supplemented with 10% sucrose and adjusted to the same pH served as the negative control.

#### 2.6.3 Cytotoxicity and viability assessment

Cytotoxicity of yeast-derived CFS preparations was evaluated in Caco-2 cells using a lactate dehydrogenase (LDH) release assay (Roche, cat. no. 11644793001, Roche, Basel, Switzerland). Caco-2 cells were exposed to CFS samples (1:10 dilution) for 24 h. Subsequently, 50 μL of cell culture supernatant was mixed with an equal volume of LDH reaction mixture and incubated for 30 min at room temperature in the dark. Absorbance was measured at 490 nm using 620 nm as the reference wavelength. Cytotoxicity percentages were calculated according to the manufacturer’s instructions. Cell viability was further assessed using the MTT assay (Roche, cat. no. 475989). Following 24 h exposure to CFS samples, 10 μL of MTT reagent was added to each well and plates were incubated for 4 h at 37 °C. Formazan crystals were dissolved overnight in 100 μL of solubilization buffer, and absorbance was measured at 570 nm with a reference wavelength between 630 and 690 nm. Viability was expressed as percentage relative to untreated controls. Cell viability was additionally confirmed using the LIVE/DEAD Viability/Cytotoxicity Kit for mammalian cells (Invitrogen, Thermo Fisher Scientific; Cat. No. L3224). Cells were incubated with staining solution containing 2 μM calcein AM and 4 μM ethidium homodimer-1 for 30 min at room temperature in the dark. Representative fluorescence micrographs were acquired using a Zeiss Axiocam 305 mono camera equipped with a 1.0× camera adapter and a 10× objective.

#### 2.6.4 Scratch wound-healing assay

The wound-healing potential of yeast-derived CFS was evaluated using an *in vitro* scratch assay following the method described by Liang et al. (2007) with minor modifications. Briefly, Caco-2 cells were cultured in complete medium until reaching approximately 90% confluence in 24-well plates. A linear scratch was generated across the cell monolayer using a sterile pipette tip, and detached cells were removed by washing with phosphate-buffered saline (PBS). Cells were then exposed to CFS (1:10 dilution in cell culture media) preparations and incubated at 37 °C under 5% CO₂. Wound closure was monitored by phase-contrast microscopy at 0 and 24 h, and images were analyzed to determine the percentage of wound closure relative to the initial scratch area. Untreated cells cultured in fresh medium served as the control. Images of the wound area were acquired immediately after scratching (0 h) and after 24 h of incubation using an inverted microscope. Wound closure was quantified using ImageJ software (National Institutes of Health, Bethesda, MD, United States). The wound area was manually outlined and measured at each time point. The percentage of wound closure was calculated according to the following formula: Wound closure (%) = [(*A*₀ − *A*₂₄)/*A*₀] × 100, where *A*₀ represents the wound area at 0 h and *A*₂₄ represents the wound area after 24 h of treatment. Experiments were performed in triplicate.

### 2.7 Statistical analysis

Data are presented as the mean ± standard deviation (SD). Each experiment was performed using three independent biological replicates, with measurements recorded in technical triplicate where applicable. Statistical analysis was conducted using GraphPad Prism version 10 (GraphPad Software, San Diego, CA, United States). Data normality was assessed using the Shapiro–Wilk test. Datasets that met the assumptions of normality were analyzed using one-way ANOVA followed by Tukey’s multiple comparisons test, whereas datasets that did not satisfy normality were analyzed using the Kruskal–Wallis test followed by Dunn’s multiple comparisons test. Statistical significance was determined at a confidence level of *p* < 0.05.

## 3 Results and discussion

### 3.1 Yeasts taxonomy and phylogenetic relationship

**Figure 1:**
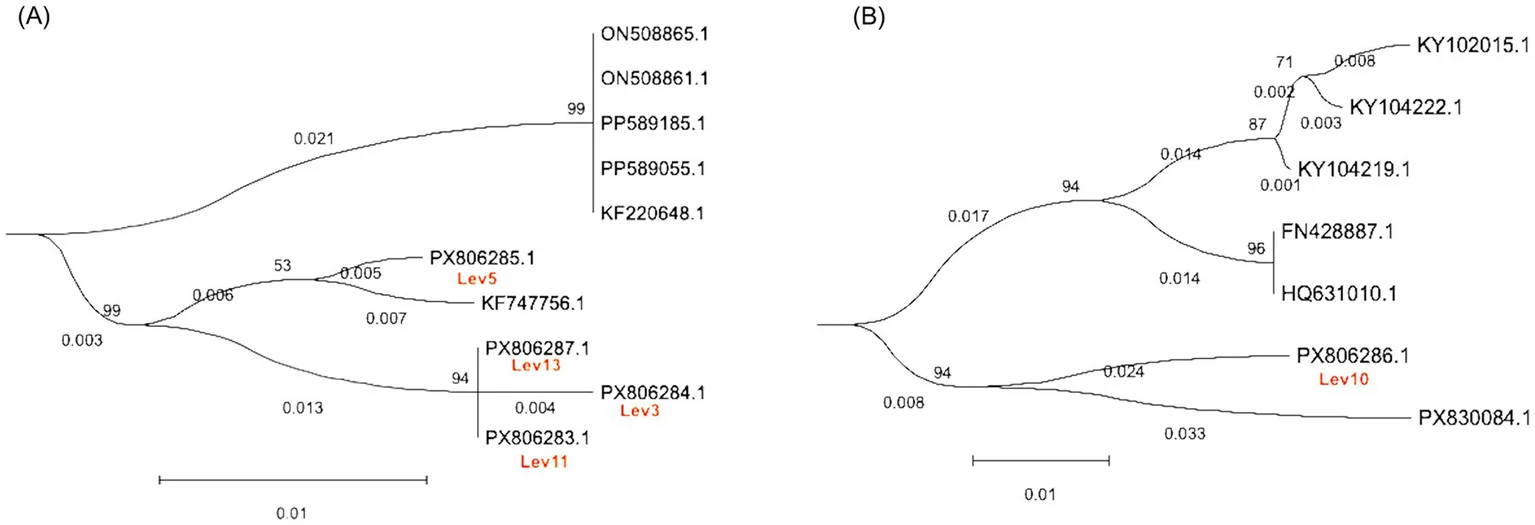
Phylogenetic relationships of yeast isolates obtained from coffee fermentation based on ITS-rRNA gene sequences. Branch support was evaluated by bootstrap analysis with 1,000 replicates, and only values above 50% are shown at the nodes. **(A)** Isolates PX806284, PX806287, PX806283, and PX806285 cluster within the *Kurtzmaniella quercitrusa* clade, forming two closely related subclades, and the tree is rooted using *Hyphopichia* sp. (ON508865) as an outgroup. **(B)** The isolate PX806286 (Lev10, Ecuador) clusters within the *Meyerozyma caribbica* clade alongside reference strains CBS:9966 and CBS:5674, with *Pichia caribbica* (FN428887) used as an outgroup. The scale bar represents the number of substitutions per nucleotide position.

Among the selected isolates, four were identified as *Kurtzmaniella quercitrusa* and one as *Meyerozyma caribbica* (Table 1). *K. quercitrusa* is commonly linked to insect-associated and plant-derived environments, such as decaying organic material and plant exudates (Kurtzman et al., 2011; Arrey et al., 2021), which aligns with its occurrence in coffee fermentations. Similar findings have been reported in other coffee-producing regions, where non-*Saccharomyces* yeasts play an active role in fermentation processes (Haile and Kang, 2019). NJ phylogenetic analysis based on ITS rRNA gene sequences clustered the four isolates (Lev3, Lev5, Lev11, and Lev13) within a well-supported *K. quercitrusa* clade, clearly separated from the reference sequences (Figure 1A). Short branch lengths and the presence of two closely related subclades indicate limited sequence divergence among the isolates at the ITS locus (Figure 1A). The isolate Lev10 clustered with reference strains of *M. caribbica* with strong bootstrap support, confirming its taxonomic assignment (Figure 1B). The presence of *M. caribbica* is consistent with its recognized role in coffee fermentation, where it contributes to carbohydrate metabolism and the production of volatile compounds that affect flavor (De Bruyn et al., 2017; Silva et al., 2024). Its frequent co-occurrence with other non-*Saccharomyces* yeasts and its persistence in sugar-rich environments further support its ecological relevance (Shen et al., 2025). In addition, its use as a starter culture has been associated with improved sensory characteristics, particularly fruity and floral notes (Evangelista et al., 2014).

**Table 1:** Taxonomic identification of yeast strains isolated from fermented cherries of different *Coffea arabica* varieties.

Group origin	Description	Sequence ID	Identification	Sample code	#Accession
*Coffea arabica* var. Typica	fermented green cherries	TYPVLev13	*Kurtzmaniella quercitrusa*	Lev13	PX806287
	fermented red cherries	TYPRLev10	*Meyerozyma caribbica*	Lev10	PX806286
*C. arabica* var. Yellow Caturra	fermented green cherries	CRYVLev5	*Kurtzmaniella quercitrusa*	Lev5	PX806285
	fermented yellow cherries	CRYALev3	*Kurtzmaniella quercitrusa*	Lev3	PX806284
*C. arabica* var. Red Caturra	fermented red cherries	CATRLev11	*Kurtzmaniella quercitrusa*	Lev11	PX806283

### 3.2 Yeasts probiotic-associated traits

#### 3.2.1 Strain-dependent adaptation of yeasts to gastrointestinal stress conditions

**Figure 2:**
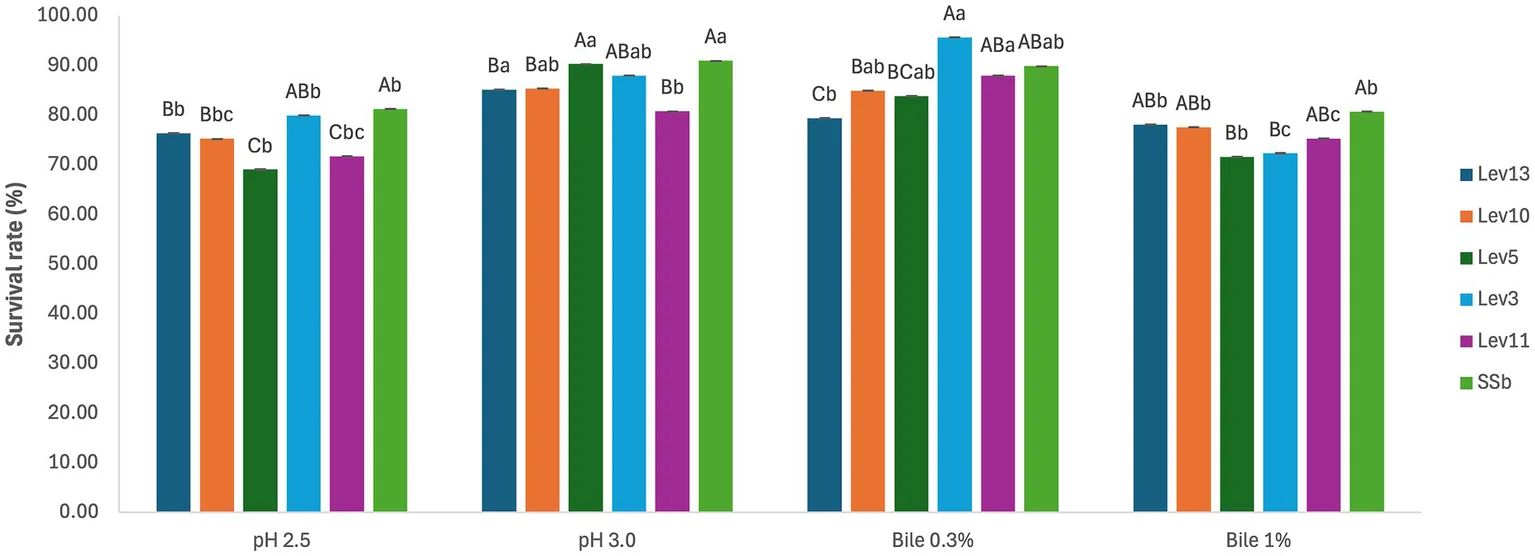
Survival rate (%) of yeast isolates after 4 h exposure to acidic (pH 2.5 and pH 3.0) and bile (0.3 and 1%) stress conditions. Values are presented as mean ± standard deviation (*n* = 3 independent replicates). Different uppercase letters above the bars indicate statistically significant differences among strains within the same treatment condition, whereas different lowercase letters indicate statistically significant differences among the four treatment conditions for the same strain (Kruskal–Wallis test followed by Dunn’s multiple comparisons test, *p* < 0.05). Bars sharing the same letter are not significantly different.

The survival rates of the tested strains varied significantly under the simulated gastrointestinal conditions (Figure 2), with significant differences among strains observed within each treatment (*p* < 0.05). Overall, all isolates maintained high viability, with survival rates exceeding 68% under all conditions. Survival was generally lower at pH 2.5 than at pH 3.0, reflecting the greater inhibitory effect of severe acid stress. At pH 2.5, Lev3 exhibited significantly higher survival than the other test isolates and was statistically comparable to the reference strain (SSb). At pH 3.0, all isolates showed improved survival, with Lev3, Lev5, and Lev10 exhibiting survival rates comparable to the reference strain. Under 0.3% bile, Lev3 achieved the highest survival (95.1%), significantly exceeding both the reference strain and the remaining isolates. Increasing the bile concentration to 1% resulted in a moderate reduction in survival across all strains; however, Lev3 and Lev10 maintained survival comparable to the reference strain. Overall, these findings demonstrate strain-dependent variation in gastrointestinal stress tolerance, with Lev3 showing the greatest overall tolerance across the tested conditions and Lev10 exhibiting consistently high survival, identifying these two isolates as the most promising candidates for further probiotic characterization. Such variability is consistent with previous studies showing that tolerance to gastrointestinal-like conditions is highly strain-dependent among yeasts (Alkalbani et al., 2022a; Zheng et al., 2024). Similar observations have been reported for *Saccharomyces boulardii*, whose resistance varies according to environmental conditions (Czerucka et al., 2007), as well as for non-conventional yeasts including *Zygosaccharomyces rouxii*, *Schizosaccharomyces pombe*, *Metschnikowia chrysoperlae*, and *Meyerozyma caribbica* (Rodríguez Machado et al., 2024; Vu Thi Thanh and Ton That Huu, 2025).

#### 3.2.2 Sodium chloride and temperature tolerance

**Figure 3:**
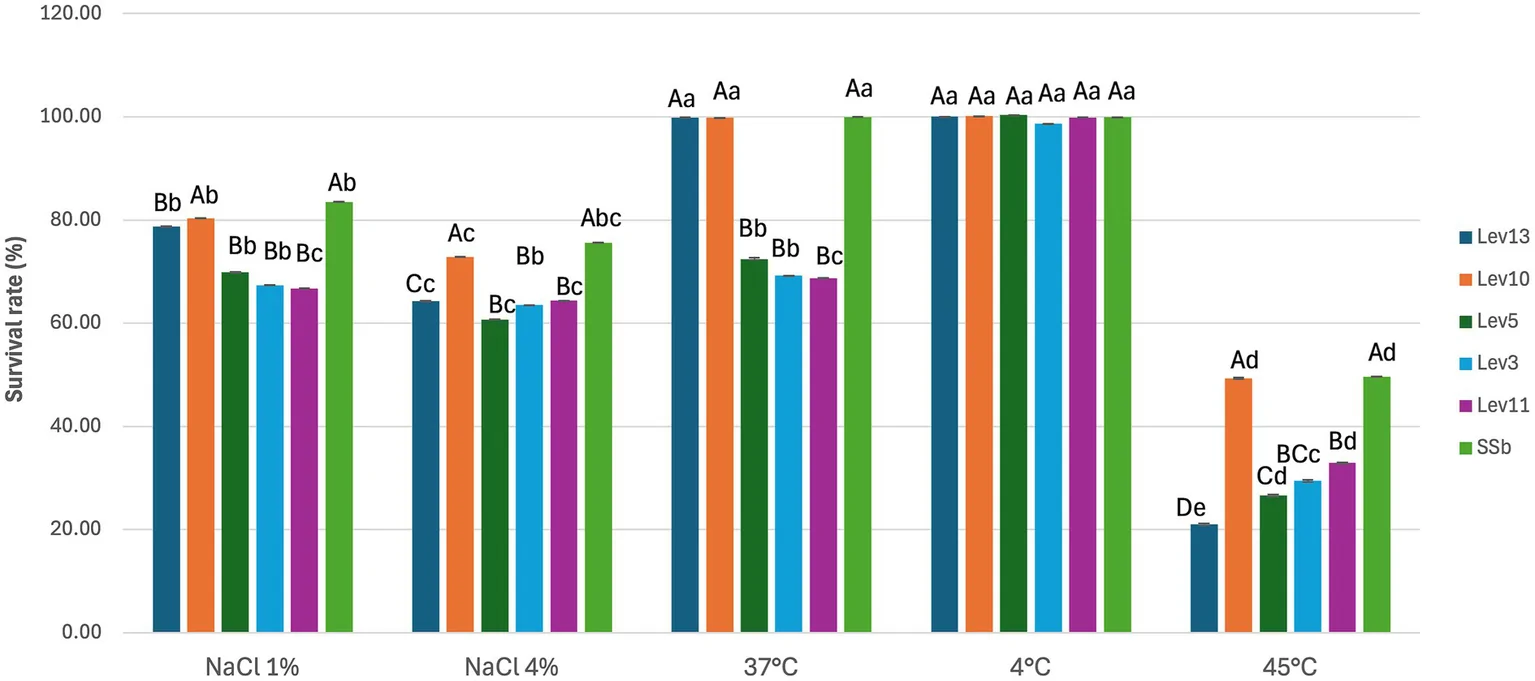
Survival rate (%) of yeast isolates after 24 h under osmotic and thermal stress conditions. Data are presented as mean ± standard deviation (*n* = 3 independent biological replicates). Different uppercase letters above the bars indicate significant differences among strains within each treatment, whereas different lowercase letters indicate significant differences among treatment conditions within the same strain (Kruskal–Wallis test followed by Dunn’s multiple comparisons test, *p* < 0.05). Bars sharing the same letter are not significantly different.

The survival rates of the isolates under osmotic and thermal stress are presented in Figure 3. Statistical analysis revealed significant differences among the strains across the treatment conditions (*p* < 0.05). All isolates maintained relatively high survival at 1% NaCl (66.2–82.7%) and 4 °C (98.3–100%). Increasing NaCl concentration to 4% reduced survival in all strains, although Lev10 retained the highest survival among the test isolates and was statistically comparable to the reference strain (SSb). At 37 °C, complete survival was observed for Lev13 and Lev10, whereas Lev5, Lev3, and Lev11 showed significantly lower survival. Exposure to 45 °C caused the greatest reduction in viability, with survival ranging from 20.8 to 48.9%. Under this condition, Lev10 exhibited significantly higher survival than the other test isolates and remained comparable to the reference strain. These results highlight the strain-dependent nature of environmental tolerance, with Lev10 emerging as a highly resilient candidate capable of withstanding both osmotic and high-temperature conditions. The tolerance to NaCl observed in the evaluated yeast isolates is consistent with previous reports describing osmoadaptation mechanisms in halotolerant yeasts. Yeast survival under saline conditions may involve activation of the high-osmolarity glycerol (HOG) pathway, intracellular accumulation of glycerol, and regulation of ion transport systems that maintain osmotic balance and membrane integrity under hyperosmotic stress (Hohmann, 2002). Similar strain-dependent variability in salt tolerance has been reported in food-associated and halotolerant yeasts, where adaptation to NaCl is linked to differential regulation of osmolyte production and stress-response pathways (Stratford et al., 2019). The better adaptation of the Lev isolates to low-temperature environments may reflect differences in membrane adaptation mechanisms (Guyot et al., 2015). Besides, SSb likely possesses heat-adaptive mechanisms possibly related to enhanced heat stress responses (Wang et al., 2025; Zielińska et al., 2025).

#### 3.2.3 Hydrophobicity and autoaggregation

**Figure 4:**
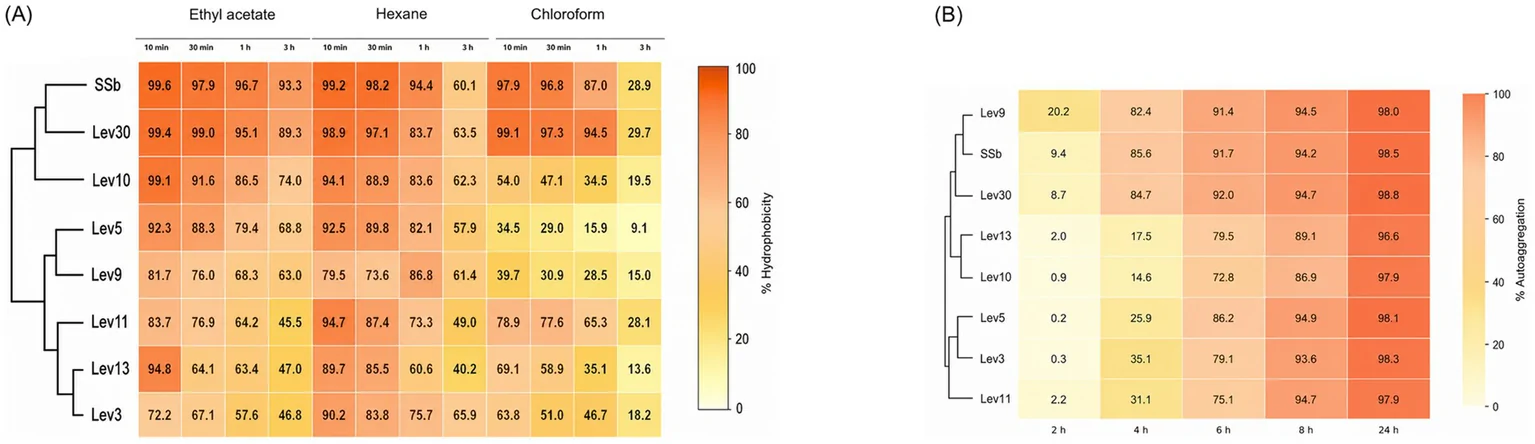
Adhesion-related properties of coffee-derived NCYs and reference strains. **(A)** Hierarchical clustering heatmap of CSH (%) determined by the microbial adhesion to hydrocarbons assay using ethyl acetate, hexane, and chloroform after 10 min, 30 min, 1 h, and 3 h. **(B)** Hierarchical clustering heatmap of autoaggregation (%) over 24 h. In both panels, cell values represent the mean of three independent biological replicates, and strains were clustered using Euclidean distance to group isolates with similar adhesion-related profiles. The color scale ranges from white (low values) to orange (high values).

Cell surface hydrophobicity (CSH), which is commonly used as an indicator of microbial surface interaction properties (Shazad et al., 2025), varied according to both the solvent and the yeast strain (Figure 4A). Hierarchical clustering based on Euclidean distance grouped strains according to the overall similarity of their hydrophobicity profiles across the three solvents and sampling times. The reference strain SSb formed a distinct cluster, reflecting its overall hydrophobicity profile across the evaluated solvents and sampling times. Lev30 clustered closely with SSb, whereas Lev10 and Lev5 formed a separate subgroup characterized by high affinity toward ethyl acetate and hexane. Lev13 and Lev11 showed intermediate profiles, while Lev3 and Lev9 clustered together due to their comparatively lower overall hydrophobicity. Across the evaluated strains, hydrophobicity was generally greatest in ethyl acetate and hexane, whereas lower values were observed with chloroform, indicating differences in cell surface physicochemical properties among isolates (Supplementary Table S2). Overall, Lev10 and Lev5 exhibited the most favorable hydrophobicity profiles among the coffee-derived isolates, suggesting more favorable adhesion-related surface characteristics than the remaining NCYs. These findings are consistent with previous reports that yeast hydrophobicity is strain-dependent (Alkalbani et al., 2022b). High CSH suggests enhanced adhesion, biofilm formation, and competitive fitness in microbial ecosystems, supporting the potential of isolates Lev10 and Lev5 for applications in fermentation and biocontrol. Overall, the observed variability underscores the importance of strain-level selection when targeting adhesion-related probiotic-associated traits among NCYs. While survival under gastrointestinal conditions is essential, the ability of yeasts to adhere to host surfaces remains a key determinant of probiotic-associated traits (Shazad et al., 2025). All NCYs exhibited a progressive increase in autoaggregation over time, with clear strain-dependent differences in aggregation kinetics (Figure 4B and Supplementary Table S3). During the first 2–6 h, Lev5, Lev3, and Lev11 aggregated more rapidly than Lev10 and Lev13, whereas the reference strains SSb, Lev30, and Lev9 exceeded 80% autoaggregation within 4 h. By 8 h, all coffee-derived isolates reached high aggregation levels (>86%), and after 24 h, all strains exhibited comparable autoaggregation (96.6–98.8%), indicating that the primary variation was in the rate rather than the final extent of aggregation. Hierarchical clustering based on Euclidean distance reflected these kinetic differences, separating the rapidly aggregating reference strains from the coffee-derived NCYs, with Lev10 and Lev13 showing the most similar profiles and Lev3 and Lev11 forming a closely related subgroup. Lev5 occupied an intermediate position, consistent with its relatively rapid early aggregation. These results indicate that autoaggregation is strain dependent, although all isolates ultimately achieved levels comparable to the probiotic reference strain (Alkalbani et al., 2022b). High autoaggregation has been associated with enhanced biofilm formation, colonization, and competitive exclusion of pathogens (Isenring et al., 2021). CSH is often positively correlated with aggregation behavior, as both are influenced by cell wall composition and surface properties (Wu et al., 2022). Consistent with this, Lev10 and Lev5 exhibited both high hydrophobicity and strong autoaggregation, suggesting favorable adhesion-related surface properties. In contrast, although Lev3 exhibited comparatively lower hydrophobicity and slower aggregation kinetics, adhesion may also involve alternative mechanisms, such as electrostatic interactions or specific adhesins (Alkalbani et al., 2022a). Overall, these findings demonstrate marked strain-dependent variation in adhesion-related properties among the evaluated NCYs. While Lev10 and Lev5 combined high hydrophobicity with strong autoaggregation, other isolates displayed distinct surface interaction profiles, emphasizing that probiotic-associated adhesion characteristics are multifactorial and strain-specific.

#### 3.2.4 Catalase and protease activity

Catalase plays a central role in decomposing hydrogen peroxide into water and oxygen, thereby preventing the accumulation of reactive oxygen species (ROS) and protecting cellular macromolecules from oxidative damage (Gómez et al., 2019). All isolates exhibited positive catalase activity, evidenced by immediate oxygen evolution upon hydrogen peroxide exposure (Supplementary Figure S1A). This uniform response indicates a conserved oxidative stress defense mechanism, which is essential for maintaining cellular redox homeostasis under gastrointestinal and fermentation-associated stress conditions. Recent studies confirm that catalase-positive yeasts exhibit enhanced resilience and technological performance, particularly among non-*Saccharomyces* species, where higher catalase activity correlates with improved stress tolerance and viability during fermentation processes (Garofalo et al., 2020). Moreover, catalase activity is increasingly recognized as a potentially beneficial stress-response trait, contributing to antioxidant capacity and facilitating survival under gastrointestinal oxidative stress, thereby may contributing to host health and microbial persistence (Yang et al., 2025; Antimanon et al., 2026). Many yeast species, particularly within *Saccharomyces* and *Candida*, are known to produce extracellular proteases with important biotechnological and pathogenic roles (Ogrydziak, 1993). In the present study, the NCYs exhibited variable protease activity based on the qualitative plate assay (Supplementary Figure S1B). The reference strains (e.g., SSb, Lev9, and Lev30) showed clear zones of hydrolysis, confirming active protease secretion. Notably, Lev10 also displayed a pronounced zone of clearing, indicating strong proteolytic activity, comparable to the reference strains. In contrast, Lev11, Lev3, and Lev5 showed moderate substrate degradation, while Lev13 exhibited weak or negligible hydrolysis. These observations demonstrate that proteolytic capacity is strain-dependent among NCYs, rather than uniformly expressed. Similar variability has been reported in non-*Saccharomyces* genera such as *Meyerozyma*, *Pichia*, and *Hanseniaspora*, where extracellular enzyme production differs across strains and environmental conditions (Buzzini et al., 2012; Padilla et al., 2016). The isolates exhibiting strong proteolytic activity suggest a potential role in protein degradation and nitrogen turnover during fermentation, potentially enhancing free amino nitrogen availability and aroma precursor formation, whereas weakly active strains may rely on alternative nitrogen sources or metabolic pathways. Further analyses of free amino nitrogen, amino acid profiles, and volatile compounds are needed to validate these functional roles.

### 3.3 Biosafety of NCYs

#### 3.3.1 Hemolytic activity

Hemolysis is considered a key virulence factor because iron acquisition is essential for microbial survival in the host, where free iron is limited and must be obtained from hemoglobin via hemolysin-mediated mechanisms (Luo et al., 2001). In this study, none of the NCY isolates showed hemolytic activity on blood agar, except the reference *Candida* strain Lev9 (Supplementary Figure S2). Hemolytic responses in yeasts are typically classified as alpha, beta, or gamma, although these categories are mainly descriptive and their mechanisms are not fully understood. In a recent study it has been shown that *M. caribbica* did not present hemolytic activity (Vu Thi Thanh and Ton That Huu, 2025). Furthermore, recent safety assessment indicates non-hemolytic activity of *K. quercitrusa* (Arrey et al., 2021; Liao et al., 2025). Thus, the lack of hemolytic activity in NCY isolates suggests a reduced virulence potential and supports their preliminary biosafety profile (Luo et al., 2001).

#### 3.3.2 Inhibition by antifungal agents

The tested yeast isolates showed variable inhibition zones, relevant for evaluating their potential safety and technological applicability (Supplementary Table S4). Most isolates exhibited clear inhibition zones in response to fluconazole and nystatin, indicating sensitivity under the tested conditions. In contrast, amphotericin B and voriconazole produced little or no detectable inhibition, suggesting reduced sensitivity at the tested concentrations. Fluconazole exhibited the strongest inhibitory activity overall, with inhibition zones exceeding 25 mm in several isolates. In contrast, Lev9 exhibited low sensitivity to fluconazole under the tested conditions, indicating reduced susceptibility to azole antifungals. This observation is relevant from a probiotic safety perspective (FAO/WHO, 2002), since antifungal-resistant *Candida* strains may represent a potential opportunistic risk and should be carefully evaluated before considering biotechnological or probiotic applications. Conversely, the remaining susceptible isolates may represent safer candidates for further functional characterization. The activity of nystatin against all strains is consistent with its membrane-targeting mechanism through ergosterol binding (Diguță et al., 2022; Ngece et al., 2024). Antifungal susceptibility profiling is considered an important criterion in the safety assessment of yeasts exhibiting probiotic-associated properties and non-conventional microbial candidates intended for food or biomedical use (Kumura et al., 2004; EFSA Panel on Dietetic Products, Nutrition and Allergies [NDA], 2011).

### 3.4 Biological activity of NCY-derived CFS

#### 3.4.1 Antimicrobial activity

The antimicrobial activity of neutralized NCY-derived CFS varied according to both the producing yeast strain and the target bacterial species (Supplementary Figure S3). Overall, all coffee-derived NCYs inhibited both Gram-negative and Gram-positive bacteria, although inhibition zones were generally moderate (approximately 8–11 mm). Gram-positive bacteria tended to be more susceptible than Gram-negative bacteria, particularly *Staphylococcus saprophyticus*, whereas activity against Gram-negative bacteria was more uniform across the tested isolates. Statistical analysis (Kruskal–Wallis followed by Dunn’s multiple comparisons test) confirmed significant strain-dependent differences for several bacterial species (Supplementary Figure S3). Because the CFS samples were neutralized to pH 7.0 before testing, the observed antimicrobial activity cannot be attributed solely to organic acids, suggesting the contribution of other extracellular antimicrobial metabolites. The bioactive compounds responsible for this activity were not characterized in the present study. Previous studies have shown that NCYs can produce diverse antimicrobial compounds, including killer toxins, siderophores such as pulcherriminic acid, hydrolytic enzymes, and volatile organic compounds that inhibit competing microorganisms (Oro et al., 2014; Melvydas et al., 2020). Further biochemical and metabolomic characterization will be necessary to identify the active compounds and clarify their mechanisms of action. Although the inhibition zones were relatively limited, the consistent inhibitory activity observed against both Gram-positive and Gram-negative bacteria suggests that these yeast isolates merit further investigation as potential sources of natural antimicrobial agents.

#### 3.4.2 Cytotoxicity and cell viability assessment

**Figure 5:**
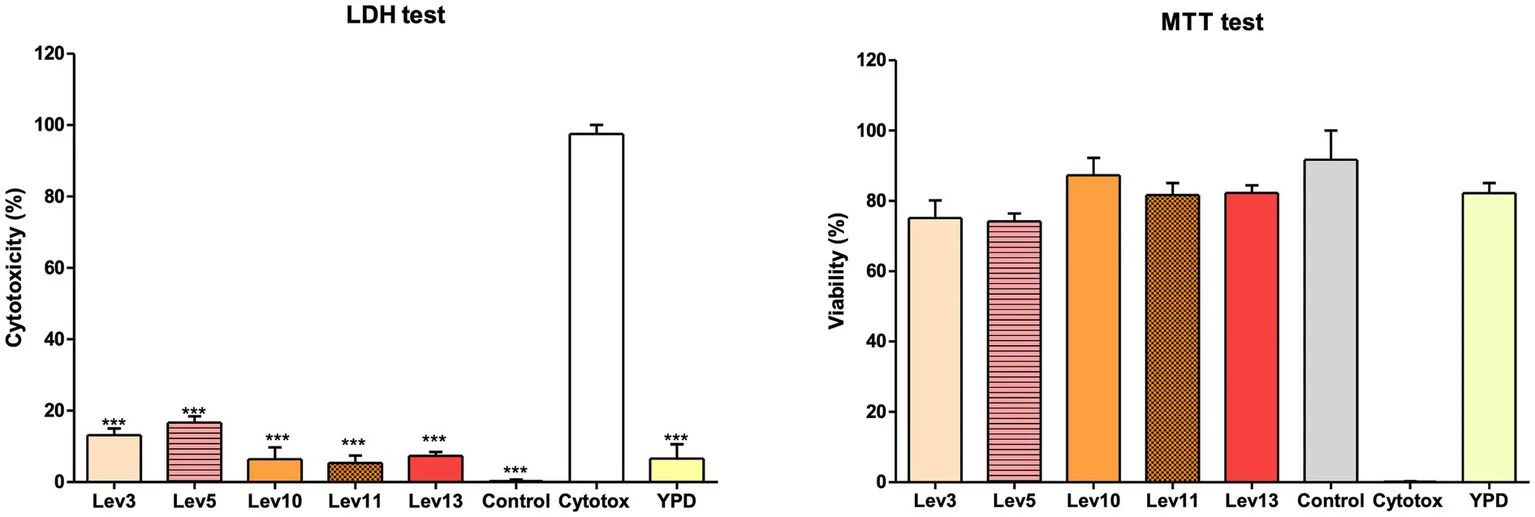
**(A)** LDH cytotoxicity assay. Cytotoxicity was determined by lactate dehydrogenase (LDH) release and expressed as a percentage relative to the positive control (Cytotox/Triton X), defined as 100% cytotoxicity. **(B)** MTT cell viability assay. Cell viability was expressed as a percentage relative to the untreated control (100% viability), whereas the cytotoxic control (Cytotox) represented minimal cell viability. Data are presented as mean ± standard deviation (*n* = 3 independent biological replicates). Statistical significance was determined by one-way ANOVA followed by Tukey’s multiple comparisons test. Asterisks indicate significant differences compared with the untreated control (***p < 0.001).

**Figure 6:**
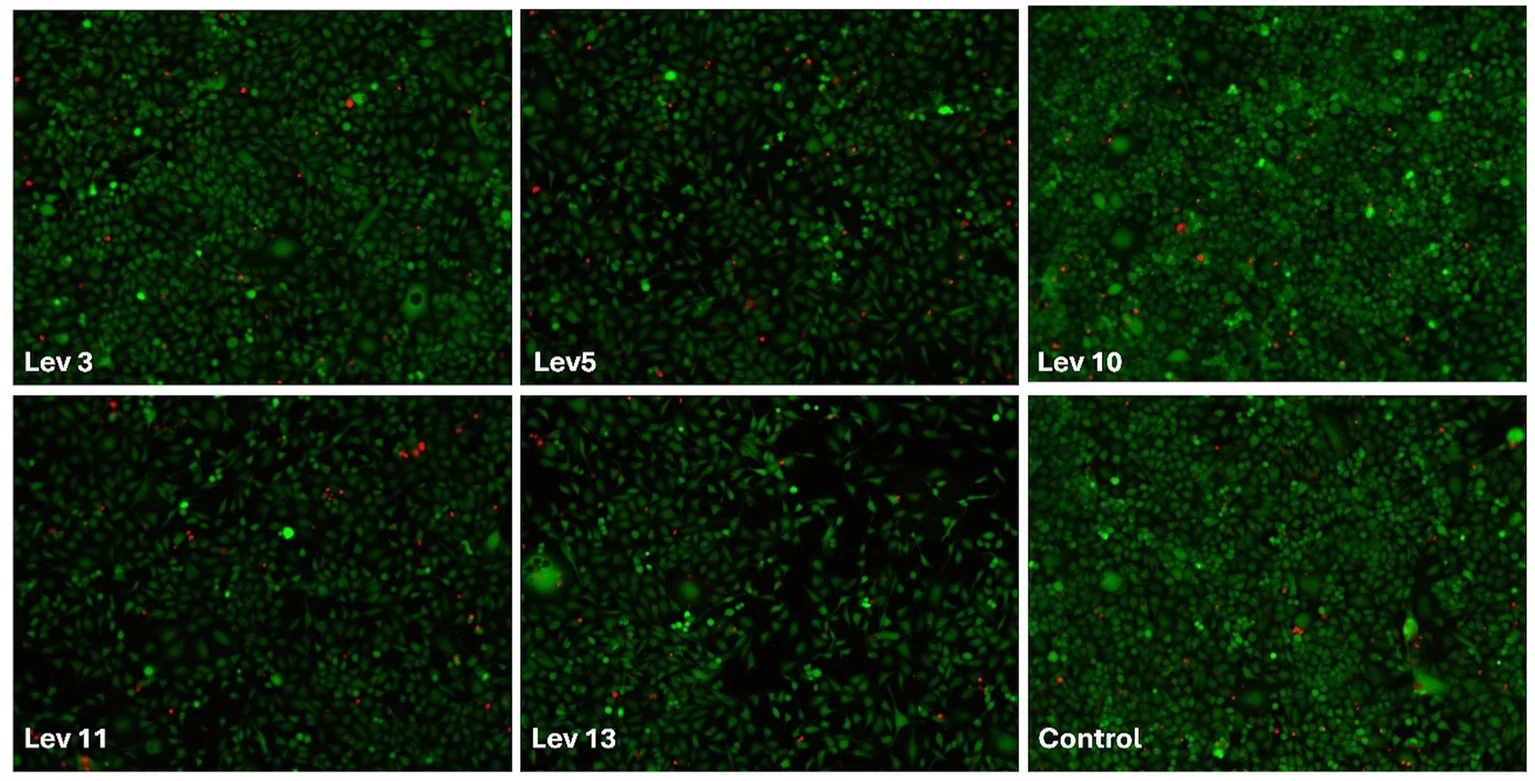
Live/Dead fluorescence staining of cells treated with Lev samples. Representative fluorescence micrographs of cells stained using the Live/Dead viability assay following exposure to the Lev samples. Green fluorescence indicates viable cells with intact membranes, whereas red fluorescence corresponds to cells with compromised membrane integrity.

The LDH assay revealed that all tested NCY-derived CFS samples induced low levels of cytotoxicity, indicating minimal membrane damage under the experimental conditions (Figure 5A). Among the strains, Lev5 exhibited the highest cytotoxicity, followed by Lev3, whereas Lev10, Lev11, and Lev13 showed consistently lower levels. Importantly, even the highest LDH release observed remained substantially below that of the positive lysis control, which approached complete cytotoxicity, confirming that overall membrane disruption was limited. These results suggest that the tested strains are generally well tolerated at the evaluated concentrations. The low LDH release in Lev10, Lev11, and Lev13 indicates preservation of membrane integrity, while the slightly higher values observed for Lev3 and particularly Lev5 may reflect a mild cytotoxic effect, although still far from levels associated with severe cellular damage (Figure 5A). The negative control showed negligible cytotoxicity, confirming low spontaneous LDH release, whereas the positive control validated the assay by inducing near-complete cell lysis. Consistent with these findings, the MTT assay demonstrated that all NCYs maintained high levels of metabolic activity, indicating preserved cell viability across conditions (Figure 5B). The untreated control exhibited maximal viability, while the cytotoxic control approached complete loss of viability, confirming the robustness and dynamic range of the assay. Among the tested strains, Lev10 showed the highest viability, followed by Lev13 and Lev11, whereas Lev3 and Lev5 displayed slightly reduced values. Nevertheless, differences among strains were modest, and overall viability remained well above levels associated with marked cytotoxicity. The Live/Dead fluorescence assay further confirmed the favorable cytocompatibility profile of the tested NCY-derived CFS (Figure 6). In all experimental groups, most cells displayed intense green fluorescence, indicating preserved membrane integrity and high cellular viability following exposure to the NCY-derived CFS. Only a limited number of red fluorescent cells, corresponding to membrane-compromised or dead cells, were observed across samples, supporting the low cytotoxicity profile previously identified through LDH and MTT assays. Among the tested isolates, Lev10 and Lev11 exhibited the highest proportion of viable, green-stained cells with very sparse red fluorescence, suggesting high cytocompatibility with the cellular model and minimal induction of membrane damage. The fluorescence pattern observed for these strains was highly comparable to the untreated control, indicating that exposure to these isolates did not substantially alter cell viability or morphology. These findings reinforce their potential suitability as postbiotic candidates.

#### 3.4.3 Wound healing properties

**Figure 7:**
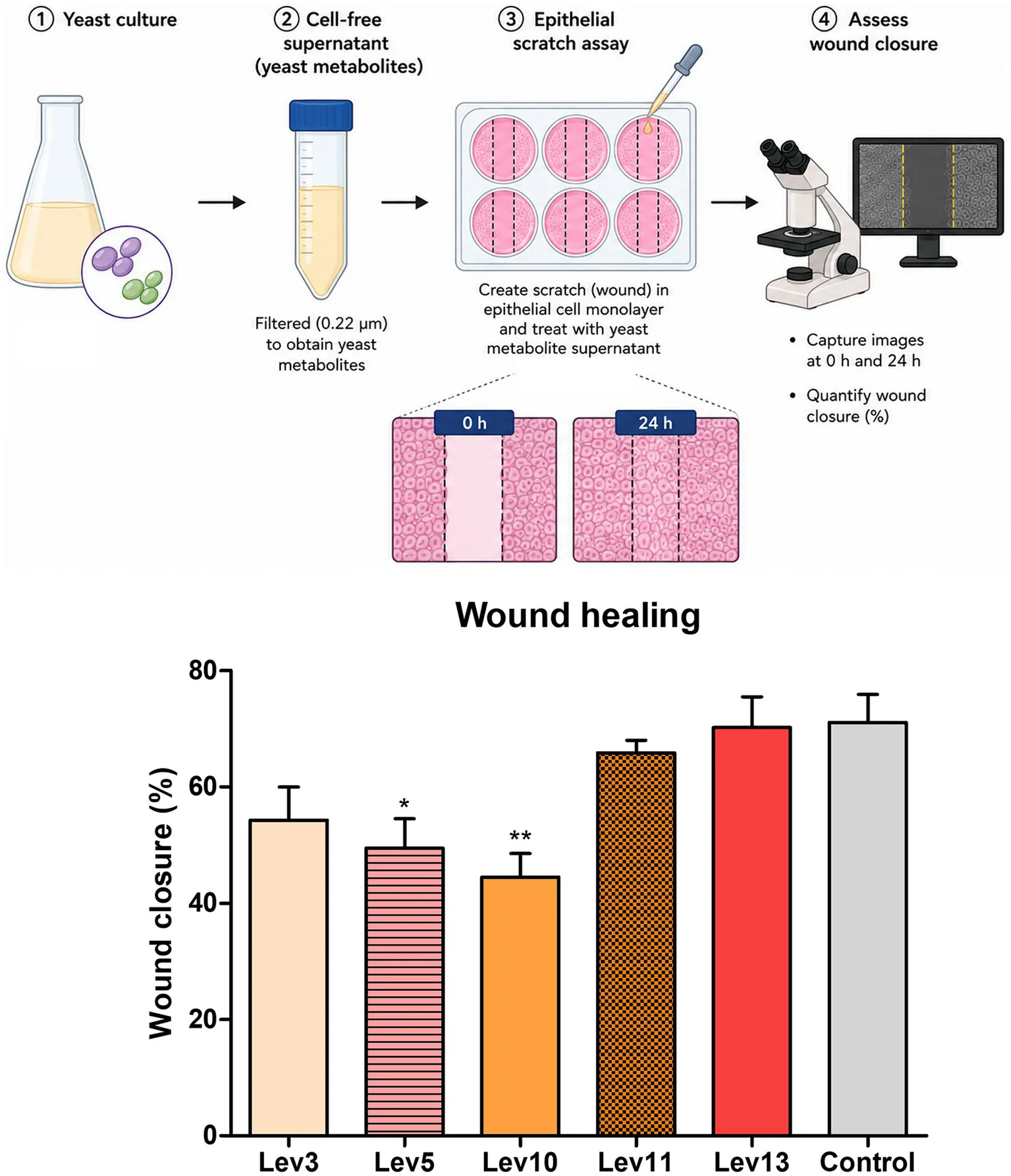
Wound healing (scratch) assay showing the effect of yeast-derived CFS on epithelial scratch closure after 24 h of treatment. Wound closure was quantified as the percentage reduction in wound area relative to the initial scratch. Data are presented as mean ± standard deviation (*n* = 3 independent biological experiments). Statistical significance was determined by one-way ANOVA followed by Tukey’s multiple comparisons test. Asterisks denote significant differences compared with the control (*p < 0.05, **p < 0.01).

Quantitative analysis of the scratch assay demonstrated strain-dependent differences in epithelial wound closure after 24 h of treatment with NCY-derived CFS (Figure 7), while representative micrographs illustrating these effects are shown in Supplementary Figure S4. Comparable initial scratch widths confirmed uniform baseline conditions across treatments. Among the evaluated isolates, Lev13 exhibited the greatest extent of scratch closure, followed by Lev11, whereas Lev3 and Lev5 displayed comparatively reduced closure. Lev11 and Lev13 maintained epithelial restitution comparable to the untreated control, while Lev10 produced an intermediate response. In contrast, wider residual wound areas and less cohesive wound fronts were observed following treatment with Lev3 and Lev5. Moreover, rounded and partially detached cells were evident in the Lev3-treated cultures, consistent with the moderate cytotoxicity detected in the LDH assay and suggesting a modest effect of this CFS on epithelial integrity. Overall, the results indicate that NCY-derived CFS exert strain-dependent effects on epithelial scratch closure without completely inhibiting wound repair or causing extensive cell detachment, supporting their general biocompatibility. The greater scratch closure observed for Lev13 and Lev11 suggests that these isolates produce extracellular metabolites that may contribute to epithelial restitution under the experimental conditions (Maurício et al., 2024). However, because the scratch assay does not distinguish between cell migration and proliferation, the mechanisms underlying wound closure cannot be established from the present data. Furthermore, only a single CFS concentration (1:10 dilution) was evaluated; therefore, dose-dependent effects remain to be determined in future studies together with *in vivo* validation. Comparable epithelial-protective effects have been reported for the probiotic yeast *S. boulardii* CNCM I-745, which contributes to intestinal barrier integrity and epithelial regeneration (Terciolo et al., 2019). Collectively, these findings indicate that selected coffee cherry-derived NCYs produce extracellular metabolites that may contribute to epithelial restitution and support their further investigation particularly as postbiotic candidates, while the probiotic potential of the producing strains requires further validation. Taken together, the cytocompatibility and scratch assay results demonstrate that the evaluated NCYs exhibit desirable characteristics for next-generation probiotic development. NCYs have recently attracted increasing attention because of their ability to tolerate harsh environmental conditions, produce bioactive metabolites, modulate host immune responses, and contribute to intestinal barrier homeostasis (Duysburgh et al., 2024; Calabrese et al., 2026).

### 3.5 Study limitations and future perspectives

The present study evaluated a limited collection of NCY isolates selected according to the criteria described in the Methods. Because *Meyerozyma caribbica* was represented by a single isolate (Lev10), the functional traits reported for this strain should not be extrapolated to the species level. The neutralized CFS displayed antimicrobial activity against the tested pathogens; however, the extracellular bioactive metabolites responsible for these effects were not characterized. In addition, epithelial restitution was assessed using a single concentration of CFS in an *in vitro* scratch assay, providing an initial indication of biological activity without allowing evaluation of concentration-dependent responses or validation under more physiologically relevant conditions.

Future studies should expand the diversity of coffee-associated NCY isolates included in the analysis and combine biochemical and metabolomic characterization of extracellular bioactive metabolites with functional validation to identify the compounds responsible for the observed activities.

## 4 Conclusion

Coffee cherry spontaneous fermentations represent a valuable source of non-conventional yeasts with promising functional properties. Molecular identification showed that the selected isolates belonged to *Kurtzmaniella quercitrusa* and *Meyerozyma caribbica*, two species that remain comparatively underexplored as candidates for probiotic- and postbiotic applications. The evaluated isolates exhibited marked strain-dependent variation in tolerance to simulated gastrointestinal conditions, osmotic and thermal stress, cell surface hydrophobicity, autoaggregation, and extracellular enzyme production. Among the isolates evaluated, Lev10 displayed the most balanced functional profile, combining high tolerance to environmental stresses with favorable adhesion-related characteristics and proteolytic activity, whereas Lev3 exhibited the greatest tolerance to simulated gastrointestinal conditions. The selected isolates also displayed a favorable preliminary biosafety profile, characterized by the absence of hemolytic activity, low cytotoxicity toward Caco-2 cells, and susceptibility to the antifungal agents evaluated under the tested conditions. In addition, their cell-free supernatants inhibited both Gram-positive and Gram-negative bacteria and maintained epithelial wound closure at levels comparable to the untreated control without affecting cell viability, supporting their potential as sources of bioactive postbiotic metabolites. Overall, the results identify selected coffee cherry-derived non-conventional yeast isolates as promising candidates for further investigation in functional food and related biotechnological applications. Future studies integrating whole-genome sequencing, metabolomic characterization of extracellular bioactive compounds, quantitative adhesion assays using intestinal epithelial cell models, and *in vivo* validation will be necessary to confirm safety, elucidate the mechanisms underlying the observed biological activities, and establish the probiotic- and postbiotic-associated potential of these isolates.

## Data Availability

The datasets presented in this study can be found in online repositories. The names of the repository/repositories and accession number(s) can be found in the article/Supplementary material.
