# Supplementary material for: Functional assessment of coffee cherry-derived non-conventional yeasts identifies promising candidates for probiotic and postbiotic applications
Source: Front Microbiol. 2026 Aug 12;17:1920410. doi: 10.3389/fmicb.2026.1920410 (PMC13506928; doi:10.3389/fmicb.2026.1920410)
Supplement: Supplementary file 1 [file Data_Sheet_1.docx]

**Supplementary files**

**Functional Assessment of Coffee Cherry-Derived Non-Conventional Yeasts Identifies Promising Candidates for Probiotic and Postbiotic Applications**

Victor Cifuentes^1^, Gratiela Gradisteanu Pircalabioru^2^, and Gabriela N. Tenea^1^*

^1^Biofood and Nutraceutics Research and Development Group, Faculty of Engineering in Agricultural and Environmental Sciences, Universidad Técnica del Norte, Ibarra 100105, Ecuador.

^2^Department of Botany and Microbiology, Faculty of Biology, University of Bucharest, 5050095 Bucharest, Romania

*****	Correspondence: gntenea@utn.edu.ec

**Table S1.** Initial physicochemical characteristics and yeast populations of coffee cherries from different Coffea arabica varieties and ripening stages subjected to spontaneous fermentation for six days at room temperature. Total soluble solids (TSS), pH, and titratable acidity were determined before fermentation, whereas pH, TSS, and viable yeast counts (CFU/mL) were measured after fermentation.

| **Coffee variety/ Group** | **Cherries ripe** | **Total soluble solids (TSS) of cherries juice** | **pH cherries juice** | **Acidity cherries juice (%)** | **pH fermented cherries** | **Total solucble solids** | **CFU/mL** |
| --- | --- | --- | --- | --- | --- | --- | --- |
| *C.arabica* L. var. typica / group A | immature green | 1.72 | 5.53 | 0.10 | 5.78 | 1.1 | 1.74E+06 |
|  | mature-red | 9.33 | 4.99 | 0.20 | 3.85 | 7.97 | 1.33E+07 |
| *C. arabica* L. var. caturra yellow /group B | immature green | 8.53 | 5.25 | 0.12 | 4.72 | 7.64 | 7.93E+05 |
|  | mature-yellow | 13.53 | 4.96 | 0.26 | 3.73 | 12.23 | 7.26E+06 |
| *C. arabica* L. var. caturra red/ group C | immature green | 3.01 | 5.31 | 0.17 | 5.39 | 2.6 | 0.00E+00 |
|  | mature-red | 10.73 | 5.99 | 0.29 | 3.62 | 9.06 | 8.21E+06 |

**Table S2.** Cell surface hydrophobicity (%) of coffee-associated yeast isolates evaluated using different organic solvents (ethyl acetate, hexane, and chloroform) at different contact times. Values represent the mean percentage of hydrophobicity from replicate measurements ± standard deviation (SD). Higher hydrophobicity values indicate greater affinity of yeast cells for the organic phase and potential differences in cell surface properties among strains.

| **Code** | **Incubation time (min)** | **Ethyl acetate** | | **Hexane** | | **Chloroform** | |
| --- | --- | --- | --- | --- | --- | --- | --- |
|  |  | **% Hydrophobicity (average)** | **SD** | **% Hydrophobicity (average)** | **SD** | **% Hydrophobicity (average)** | **SD** |
| **Lev13** | 10 | 94.764 | 0.002 | 89.665 | 0.001 | 69.121 | 0.002 |
|  | 30 | 64.081 | 0.002 | 85.522 | 0.003 | 58.896 | 0.001 |
|  | 60 | 63.350 | 0.001 | 60.616 | 0.002 | 35.100 | 0.002 |
|  | 180 | 47.008 | 0.001 | 40.213 | 0.003 | 13.571 | 0.002 |
| **Lev10** | 10 | 99.058 | 0.002 | 94.087 | 0.001 | 54.009 | 0.002 |
|  | 30 | 91.618 | 0.002 | 88.873 | 0.001 | 47.089 | 0.002 |
|  | 60 | 86.517 | 0.001 | 83.626 | 0.002 | 34.464 | 0.002 |
|  | 180 | 73.960 | 0.001 | 62.281 | 0.001 | 19.528 | 0.002 |
| **Lev5** | 10 | 92.309 | 0.001 | 92.490 | 0.001 | 34.498 | 0.001 |
|  | 30 | 88.291 | 0.002 | 89.756 | 0.001 | 29.027 | 0.002 |
|  | 60 | 79.397 | 0.003 | 82.131 | 0.001 | 15.856 | 0.001 |
|  | 180 | 68.791 | 0.001 | 57.938 | 0.001 | 9.085 | 0.002 |
| **Lev3** | 10 | 72.200 | 0.002 | 90.157 | 0.002 | 63.826 | 0.002 |
|  | 30 | 67.107 | 0.002 | 83.823 | 0.002 | 50.996 | 0.002 |
|  | 60 | 57.640 | 0.001 | 75.694 | 0.002 | 46.735 | 0.002 |
|  | 180 | 46.784 | 0.001 | 65.899 | 0.002 | 18.217 | 0.002 |
| **Lev11** | 10 | 83.676 | 0.002 | 94.727 | 0.002 | 78.861 | 0.001 |
|  | 30 | 76.918 | 0.002 | 87.414 | 0.001 | 77.620 | 0.001 |
|  | 60 | 64.202 | 0.002 | 73.262 | 0.001 | 65.312 | 0.002 |
|  | 180 | 45.478 | 0.002 | 49.021 | 0.001 | 28.061 | 0.001 |
| **SSb** | 10 | 99.576 | 0.001 | 99.152 | 0.001 | 97.913 | 0.001 |
|  | 30 | 97.896 | 0.001 | 98.222 | 0.001 | 96.836 | 0.001 |
|  | 60 | 96.673 | 0.001 | 94.439 | 0.001 | 87.035 | 0.001 |
|  | 180 | 93.297 | 0.001 | 60.111 | 0.000 | 28.947 | 0.001 |
| **Lev 30** | 10 | 99.420 | 0.001 | 98.872 | 0.001 | 99.132 | 0.001 |
|  | 30 | 99.038 | 0.001 | 97.148 | 0.001 | 97.331 | 0.001 |
|  | 60 | 95.058 | 0.002 | 89.701 | 0.001 | 94.466 | 0.001 |
|  | 180 | 89.254 | 0.001 | 63.466 | 0.001 | 29.699 | 0.001 |
| **Lev 9** | 10 | 81.712 | 0.001 | 79.512 | 0.002 | 39.706 | 0.002 |
|  | 30 | 75.996 | 0.001 | 73.587 | 0.001 | 30.865 | 0.002 |
|  | 60 | 68.304 | 0.002 | 86.850 | 0.002 | 28.516 | 0.001 |
|  | 180 | 63.006 | 0.002 | 61.448 | 0.002 | 14.976 | 0.001 |

**Table S3.** Autoaggregation capacity (%) of coffee-associated yeast isolates during incubation at different time points. Autoaggregation percentages were measured after 0, 2, 4, 6, 8, and 24 h of incubation and are expressed as mean values ± standard deviation (SD).

| **Code** | **Incubation time (h)** | **% Autoaggregation** | **SD** |  |
| --- | --- | --- | --- | --- |
|  |  |  |  |  |
| **Lev13** | 0 | **0.000** | 0.000 |  |
|  | 2 | **1.966** | 0.005 |  |
|  | 4 | **17.456** | 0.007 |  |
|  | 6 | **79.465** | 0.005 |  |
|  | 8 | **89.055** | 0.007 |  |
|  | 24 | **96.631** | 0.006 |  |
| **Lev10** | 0 | **0.000** | 0.000 |  |
|  | 2 | **0.898** | 0.008 |  |
|  | 4 | **14.643** | 0.006 |  |
|  | 6 | **72.788** | 0.009 |  |
|  | 8 | **86.941** | 0.006 |  |
|  | 24 | **97.911** | 0.004 |  |
| **Lev5** | 0 | **0.000** | 0.000 |  |
|  | 2 | **0.212** | 0.002 |  |
|  | 4 | **25.866** | 0.006 |  |
|  | 6 | **86.209** | 0.007 |  |
|  | 8 | **94.853** | 0.006 |  |
|  | 24 | **98.121** | 0.008 |  |
| **Lev3** | 0 | **0.000** | 0.000 |  |
|  | 2 | **0.343** | 0.006 |  |
|  | 4 | **35.065** | 0.006 |  |
|  | 6 | **79.101** | 0.006 |  |
|  | 8 | **93.595** | 0.009 |  |
|  | 24 | **98.252** | 0.008 |  |
| **Lev11** | 0 | **0.000** | 0.000 |  |
|  | 2 | **2.193** | 0.008 |  |
|  | 4 | **31.059** | 0.006 |  |
|  | 6 | **75.146** | 0.006 |  |
|  | 8 | **94.721** | 0.004 |  |
|  | 24 | **97.872** | 0.005 |  |
| **SSb** | 0 | **0.000** | 0.000 |  |
|  | 2 | **9.365** | 0.009 |  |
|  | 4 | **85.560** | 0.009 |  |
|  | 6 | **91.699** | 0.008 |  |
|  | 8 | **94.221** | 0.005 |  |
|  | 24 | **98.477** | 0.004 |  |
| **Lev30** | 0 | **0.000** | 0.000 |  |
|  | 2 | **8.650** | 0.003 |  |
|  | 4 | **84.650** | 0.009 |  |
|  | 6 | **92.033** | 0.008 |  |
|  | 8 | **94.683** | 0.008 |  |
|  | 24 | **98.797** | 0.003 |  |
| **Lev9** | 0 | **0.000** | 0.000 |  |
|  | 2 | **20.232** | 0.001 |  |
|  | 4 | **82.385** | 0.002 |  |
|  | 6 | **91.397** | 0.003 |  |
|  | 8 | **94.455** | 0.002 |  |
|  | 24 | **98.021** | 0.001 |  |

**Table S4.** Antifungal activity of yeast isolates determined by agar well diffusion assay.

| **Strain** | **Nystatin (NYS 1.25ug/mL)** | **Amphotericin**  **(AMB 2 ug/mL)** | **Fluconazole (FLU 64ug/mL)** | **Voriconazole (VRC 2ug/mL)** |
| --- | --- | --- | --- | --- |
| Lev13 | 11.8 (S) | 6 (R) | 27.1 (S) | 6 (R) |
| Lev10 | 14.9 (S) | 6 (R) | 18.7 (S) | 6 (R) |
| Lev5 | 12.7 (S) | 6 (R) | 27.8 (S) | 6 (R) |
| Lev3 | 10.9 (S) | 6 (R) | 23.9 (S) | 6 (R) |
| Lev11 | 12.4 (S) | 6 (R) | 26.9 (S0 | 6 (R) |
| SSb | 17.9 (S) | 6 (R) | 25.3 (S) | 6 (R) |
| Lev9 | 15.8 (S) | 6 (R) | 6 (R) (S) | 6 (R) |
| Lev30 | 19.4 (S) | 6 (R) | 28.3 (S) | 6 (R) |

Inhibition zone diameters are expressed in millimeters. S, susceptible; R, resistant.

**Figure S1.** Catalase and protease activity of yeasts. (A) Catalase activity assessed by bubble formation following hydrogen peroxide application, where increased bubbling indicates higher enzymatic activity. (B) Protease activity evaluated on agar plates supplemented with skim milk (10%) based on growth intensity and proteolytic halo formation along the inoculation streak. Legend: Lev10: *Meyerozyma carribica*; Lev13, Lev5, Lev3, Lev11-*Kurtzamaniella quercitrusa*; SSb: *S. boulardii* CNCM 1-745; Lev9: *Candida intermedia* Lev9; Lev30: *S. cerevisiae* Lev30

(B)

**Figure S2.** Hemolytic activity. All yeast strains exhibited no hemolytic activity (γ-hemolysis), as evidenced by the absence of clear or green halos, in contrast to the positive β-hemolytic control (Lev 9), which showed pronounced clearing, confirming their non-hemolytic and potentially safe phenotype. Legend: Lev10: *Meyerozyma carribica*; Lev13, Lev5, Lev3, Lev11-*Kurtzamaniella quercitrusa*; SSb: *S. boulardii* CNCM 1-745; Lev9: *Candida intermedia; Lev30: S. cerevisae.*

**Figure S3.** Antimicrobial activity of neutralized CFS produced by coffee cherry-derived NCYs and reference strains against Gram-negative (A) and Gram-positive (B) bacteria, determined by the agar well diffusion assay. Bars represent the mean inhibition zone diameter (mm) ± SD of three independent biological replicates. Different uppercase letters above bars indicate significant differences among yeast strains for each bacterial species according to the Kruskal–Wallis test followed by Dunn's multiple comparisons test (P < 0.05). The indicator microorganisms: Escherichia coli ATCC 25922 (A), E. coli L1PEag1 (B), Salmonella enterica ATCC 35640 (C), S. enterica ATCC 51741 (D), Shigella sonnei ATCC 25931 (E), Enterobacter sp. dFMcEag13 (F), Kluyvera intermedia dFMPEag15 (G), Ewingella sp. UMEag21 (H), Pantoea sp. UMEag23 (I), Serratia liquefaciens P4StpC1 (J), Staphylococcus aureus ATCC 1026 (K), S. aureus ATCC 43300 (L), S. epidermidis L4mStp5 (M), S. xylosus  FFCShyA4 (N), and Listeria monocytogenes ATCC 19115 (O).

(A).

(B).

**Figure S4.** Wound healing assay of epithelial cells treated with non-conventional yeast (NCY) isolates after 24 h. Representative phase-contrast microscopy images illustrating scratch closure following treatment with the Lev yeast isolates compared to the untreated control. Images were acquired 24 h after scratch induction.
